# *Tomiyamichthys oriens*, a new species of shrimpgoby (Teleostei, Gobiidae) from the coast of Banyuwangi, East Java, Indonesia

**DOI:** 10.3897/zookeys.1283.194940

**Published:** 2026-06-24

**Authors:** Kunto Wibowo, Hilda M. Pratiwi, Anik B. Dharmayanthi, Djohan Tjiptadi, Wiwi Tarmini, Agus Priyadi, Ruby V. Kusumah

**Affiliations:** 1 Museum Zoologicum Bogoriense, Research Center for Biosystematics and Evolution, National Research and Innovation Agency, Cibinong 16911, Indonesia Museum Zoologicum Bogoriense, Research Center for Biosystematics and Evolution, National Research and Innovation Agency Cibinong Indonesia; 2 CV Cahaya Baru, Jakarta Selatan 12320, Indonesia CV Cahaya Baru Jakarta Indonesia; 3 Research Center for Biota System, National Research and Innovation Agency, Cibinong 16911, Indonesia Research Center for Biota System, National Research and Innovation Agency Cibinong Indonesia; 4 Research Center for Applied Zoology, National Research and Innovation Agency, Cibinong 16911, Indonesia Research Center for Applied Zoology, National Research and Innovation Agency Cibinong Indonesia

**Keywords:** Coloration, marine ornamental fishes, meristics, mitochondrial COI, taxonomy, *Tomiyamichthys hyacinthinus*

## Abstract

The new shrimpgoby, *Tomiyamichthys oriens***sp. nov**., is described based on two specimens collected off Banyuwangi, East Java, Indonesia. The new species is most similar to the recently described Japanese species *T. hyacinthinus*, both species being distinguished from all other species of *Tomiyamichthys* by having nine segmented dorsal- and anal-fin rays and two elongated filaments (second and third spines) in the first dorsal fin of males. The new species differs from *T. hyacinthinus* in several meristic characters (including numbers of pectoral- and caudal-fin rays, longitudinal scales, and gill rakers), as well as coloration. In addition, the morphological differences were supported by mitochondrial COI analyses, which demonstrated clear genetic differentiation (divergence of 3.1%) from *T. hyacinthinus*.

## Introduction

The shrimp-associated goby genus *Tomiyamichthys* Smith, 1956 is broadly distributed throughout the tropical Indo-Pacific, extending from the Red Sea and eastern African coast to Japan and Fiji. To date, 20 species are recognized as valid (Allen et al. 2023; Sato and Motomura 2025), all being diagnosed by a characteristic suite of features: ventral one-third or more of lower gill slit closed by membrane; 11 or fewer rudimentary or relatively short gill rakers on outer surface of first gill arch; a well-developed longitudinal pattern of sensory papillae rows on cheek and uniserial transverse row of sensory papillae just behind chin; equal numbers of dorsal- and anal-fin rays; numerous small scales, often non-imbricate anteriorly; and a moderately narrow gill opening reaching to below opercle (Hoese et al. 2016).

Situated within the center of the Coral Triangle, Indonesia supports a remarkably high diversity of gobiid fishes, including 12 species of *Tomiyamichthys*, recorded through field collections and underwater photography (see Allen and Erdmann 2024), e.g., *T. alleni* Iwata, Ohnishi & Hirata, 2000 (Bali and Flores), *T. emilyae* Allen, Erdmann & Utama, 2019 (Bali and North Sulawesi), *T. eyreae* Allen, Erdmann & Mongdong, 2020 (West Papua), *T. gomezi* Allen & Erdmann, 2012 (Seribu Island of Java), *T. lanceolatus* (Yanagisawa, 1978) (eastern Indonesia), *T. latruncularius* (Klausewitz, 1974) (Java and northern Sulawesi), *T. nudus* Allen & Erdmann, 2012 (West Papua), *T. oni* (Tomiyama, 1936) (Sumatra), *T. russus* (Cantor, 1849) (Sumatra and Java), *T. smithi* (Chen & Fang, 2003) (West Papua), *T. tanyspilus* Randall & Chen, 2007 (Sumbawa and Flores), and *T. zonatus* Allen, 2015 (Flores).

The new species of *Tomiyamichthys* described herein is based on two specimens that were first brought to our attention by the ornamental fish and marine biota exporter CV Cahaya Baru and were collected off Banyuwangi, East Java, Indonesia. The species closely resembles the recently described Japanese species *T. hyacinthinus* Sato & Motomura, 2025, particularly in having nine segmented dorsal- and anal-fin rays and the second and third spines of the first dorsal fin in males being elongated and filamentous. However, it differs from the latter in several meristic and coloration characters. In addition, mitochondrial COI-based phylogenetic analyses indicated clear genetic differentiation, with a divergence of 3.1% from *T. hyacinthinus*, justifying the description of the former as a new species.

## Material and methods

The specimens described in this study were collected off Banyuwangi, East Java, Indonesia, and photographed, fixed, and preserved following Motomura and Ishikawa (2013). Counts and measurements follow Allen et al. (2020). Standard and head lengths are abbreviated as SL and HL, respectively. Measurements were taken under a stereo microscope (Olympus SZX7, Japan) to the nearest 0.01 mm, with Mitutoyo digital calipers (Model No. CD–S15C). Terminology of the cephalic sensory papillae rows follows Akihito et al. (2002). Sex was tentatively determined based on external sexual dimorphic characters, particularly the morphology of the first dorsal fin, consistent with patterns observed in congeners. The specimens are deposited in the Museum Zoologicum Bogoriense (MZB), Bogor, Indonesia. Field collection numbers are indicated in parentheses following MZB catalog numbers. In addition to the data obtained from the material examined in this study, information from previously published species descriptions and taxonomic revisions of *Tomiyamichthys* was also used for comparative purposes, including Allen et al. (2020) for *T. eyreae* and *T. latruncularius*, and Sato and Motomura (2025) for *T. hyacinthinus*.

For DNA barcoding, total genomic DNA of *T. oriens* sp. nov. and *T. oni* were extracted from muscle tissue preserved in 99% ethanol using the MagMAX Multi-Sample DNA Extraction Kit (Applied Biosystems), following the manufacturer’s protocol. A partial fragment of the mitochondrial cytochrome *c* oxidase subunit I (COI) gene was amplified using the universal fish primers FishF1 (5'-TCAACCAACCACAAAGACATTGGCAC-3') and FishR1 (5'-TAGACTTCTGGGTGGCCAAAGAATCA-3') (Ward et al. 2005). PCR amplifications were performed in a total volume of 25 μL, consisting of 12.5 μL of Taq polymerase master mix, 1 μL of each primer (forward and reverse), 1 μL of DNA template, and 9.5 μL of nuclease-free water. The thermal cycling conditions included initial denaturation at 95 °C for 2 min, followed by 35 cycles of 94 °C for 30 s, 54 °C for 30 s, and 72 °C for 1 min, with a final extension at 72 °C for 10 min, and held at 4 °C. PCR products were visualized on 1.5% agarose gels. Successful amplicons were sequenced bidirectionally using an automated DNA sequencer (ABI 3500, Applied Biosystems Inc., CA, USA) at the BRIN sequencing facility, Indonesia. The resulting sequences were edited and aligned in Geneious Prime 2025.0.3 (https://www.geneious.com). Subsequently, the sequences were deposited in GenBank (National Center for Biotechnology Information, NCBI; https://www.ncbi.nlm.nih.gov) and compared with available sequences using BLAST (Basic Local Alignment Search Tool; https://blast.ncbi.nlm.nih.gov/Blast.cgi). All sequences of two *T. oriens* sp. nov. and one *T. oni* samples were submitted to NCBI with Accession Numbers PZ466150 (holotype), PZ466151 (paratype), and PZ466149, respectively. Comparative data for seven species of *Tomiyamichthys*, deposited by Allen et al. (2023), Chan et al. (2024), and Sato and Motomura (2025), were downloaded from GenBank and used for phylogenetic reconstruction, including *T. elliotensis* (Australia: OR579086.1, OR579087.1, OR579088.1), *T. lanceolatus* (Papua New Guinea: HQ536662.1), *T. latruncularius* (Indonesia: OR579090.1, OR579092.1, OR579093.1), *T. oni* (Indonesia: OR579089.1), *T. russus* (Philippines: OQ387379.1; Singapore: PP089012.1, PP089013.1), *T. tanyspilus* (Papua New Guinea: OR579089.1), and *T. hyacinthinus* (Japan: LC841836, LC841837, LC841838). The sequence of *Stonogobiops xanthorhinica* Hoese & Randall, 1982 (HQ536655.1) (Thacker et al. 2011) was used as an outgroup. The best-fit nucleotide substitution model was selected using MEGA X (Stecher et al. 2020), and phylogenetic relationships were inferred using Bayesian inference (Huelsenbeck and Ronquist 2001).

## Results

### Tomiyamichthys oriens sp. nov.

Taxon classificationAnimaliaPerciformesGobiidae

47B6EA24-25EF-5D29-B3F5-009AC422679A

https://zoobank.org/AB73BAC3-AF1C-409B-B88D-D29D950F0ACF

**Figure 1.**
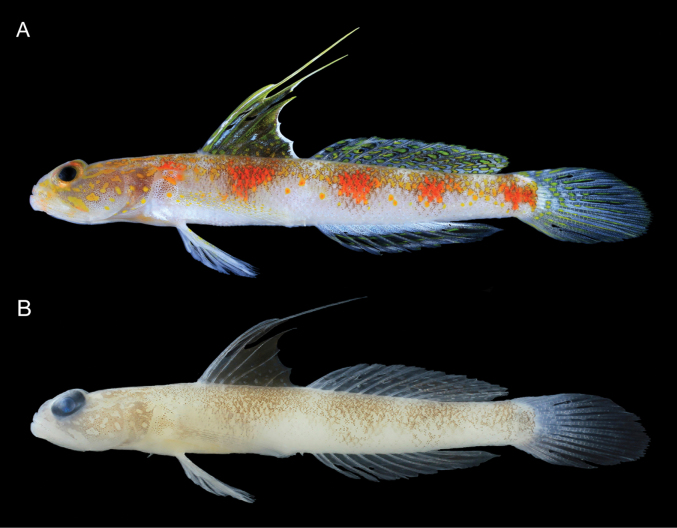
Holotype of *Tomiyamichthys oriens* sp. nov., MZB 28902, male, 35.6 mm SL, off Banyuwangi, East Java, Indonesia. **A**. Fresh specimen; **B**. Preserved specimen.

**Figure 2.**
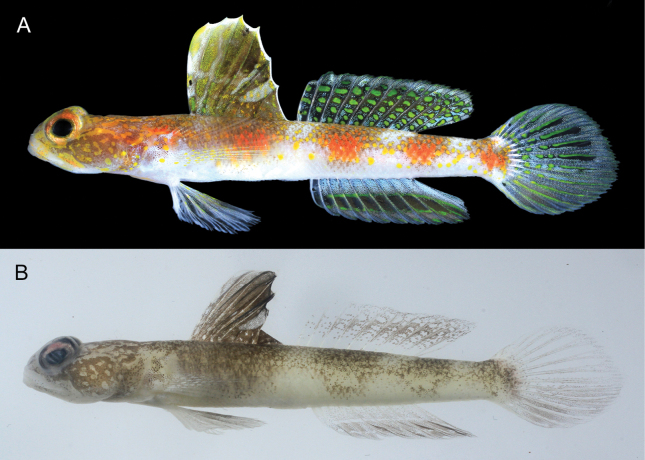
Paratype of *Tomiyamichthys oriens* sp. nov., MZB 28903, female, 35.5 mm SL, off Banyuwangi, East Java, Indonesia. **A**. Fresh specimen; **B**. Preserved specimen.

**Figure 3.**
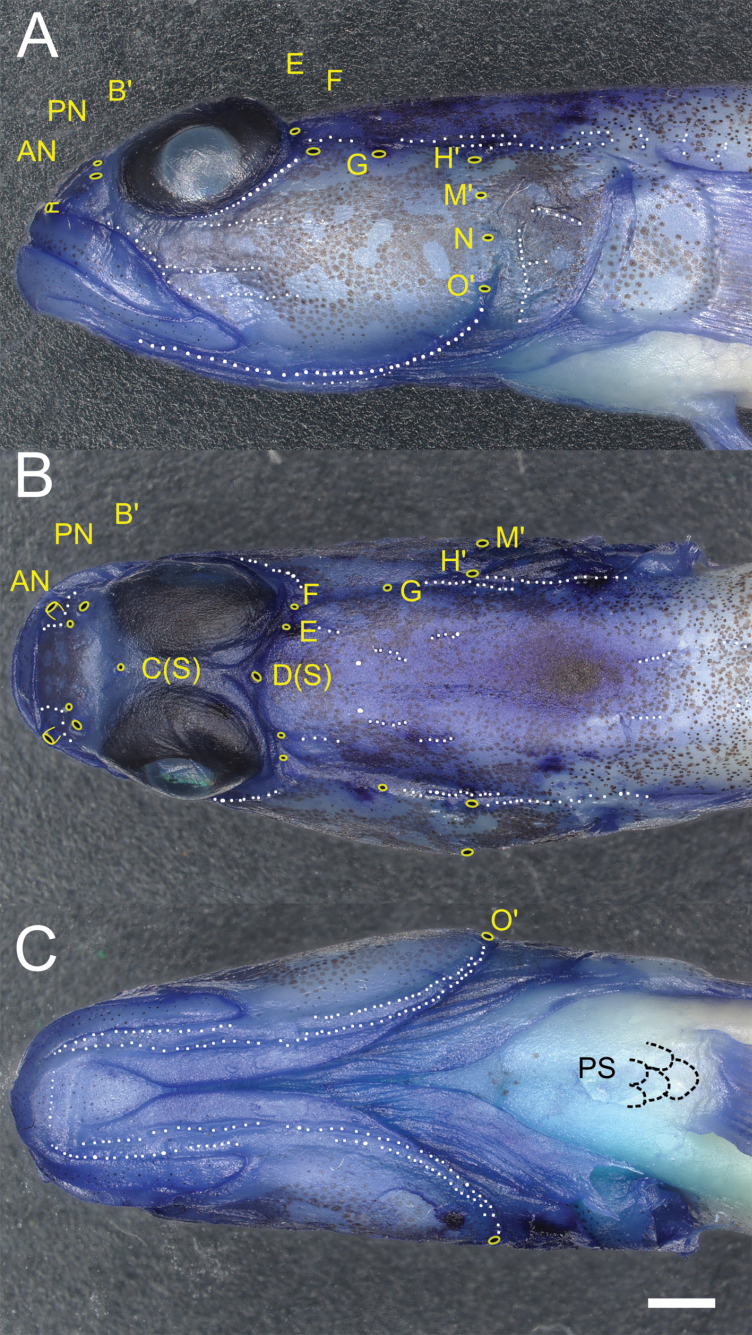
Head of holotype of *Tomiyamichthys oriens* sp. nov., MZB 28902, male, 35.6 mm SL, showing sensory papillae (white spots), pores (black spots with yellow edges), and prepelvic scales (PS) (black dashed line). **A** Lateral; **B**. Dorsal; **C**. Ventral views. Scale bar: 1 mm.

Figs 1, 2, 3, Table 1

#### New English name.

Oriens Shrimpgoby.

#### Type materials.

***Holotype*. Indonesia** • 35.6 mm SL; Java Island, East Java Province, Banyuwangi Regency, 21 Aug. 2025; obtained from CV Cahaya Baru; collected using a fine-mesh hand net; MZB 28902 (INA 1475). ***Paratype***. 35.5 mm SL; same data as holotype; MZB 28903 (INA 1580).

#### Diagnosis.

A new species of *Tomiyamichthys* with the following combination of characters: dorsal-fin rays VI-I, 9; anal-fin rays I, 9; pectoral-fin rays 18; branched caudal-fin rays 13; longitudinal scale rows 60 or 61; transverse scale rows 15; gill rakers of first gill arch 3 + 8 (including rudimentary rakers); prepelvic region with partially embedded scales; two elongated filaments (second and third spines) in first dorsal fin of males; first and fourth spines of first dorsal fin approximately equal in length in both males and females; first dorsal fin trapezoid, without filamentous spines in females; a few relatively large orange spots on lateral surface of head (size similar to spots at base of second dorsal fin); five radiant orange blotches along lateral surface of body; irregular white blotches absent on lateral body surface; and indistinct black spot on membrane between fourth and fifth spines of first dorsal fin.

#### Description.

Counts and measurements given in Table 1. Head and body compressed; head broader than deep; body elongated, deepest at pelvic-fin origin. Snout short, clearly shorter than pupil width. Eyes close set, nearly touching; interorbital space extremely narrow; eyes slightly interrupting head profile; well-defined longitudinal groove above eye, extending from interorbital to postorbital region. Cheek moderately bulged.

**Table 1.** Counts and measurements (expressed as percentages of standard length) of *Tomiyamichthys oriens* sp. nov. from East Java, Indonesia.

	Holotype	Paratype
	MZB 28902	MZB 28903
	Male	Female
Standard length (SL; mm)	35.6	35.5
Count		
First dorsal-fin rays	VI	VI
Second dorsal-fin rays	I, 9	I, 9
Anal-fin rays	I, 9	I, 9
Pectoral-fin rays	18	18
Pelvic-fin rays	I, 5	I, 5
Segmented caudal-fin rays	15	15
Branched caudal-fin rays	13	13
Upper unsegmented caudal-fin rays	c. 4	c. 4
Lower unsegmented caudal-fin rays	c. 3	c. 3
Longitudinal scale rows	60	61
Transverse scale rows	15	15
Gill rakers	3+8	3+8
Prepelvic scales embedded	partially	partially
Body scales	ctenoid posteriorly	ctenoid posteriorly
Measurement (% of SL)		
Body depth (at pelvic-fin origin)	13.7	13.9
Body depth (at anal-fin origin)	12.6	12.9
Body width	12.7	12.3
Head length	26.8	27.9
Head width	13.1	13.9
Snout length	6.3	6.1
Orbit diameter	7.4	7.2
Cheek depth	6.9	7.0
Upper-jaw length	10.9	11.6
Caudal-peduncle depth	8.8	8.7
Caudal-peduncle length	18.6	18.9
Predorsal fin length	33.5	34.4
Pre-anal fin length	57.7	59.1
Prepelvic fin length	29.0	31.0
Dorsal-fin base length	48.1	48.9
First dorsal-fin first spine length	20.2	20.6
First dorsal-fin third spine length	35.6	21.9
First dorsal-fin fourth spine length	19.8	20.3
First dorsal-fin fifth spine length	13.5	16.7
Second dorsal-fin spine length	10.5	9.9
Longest dorsal-fin ray length	19.6	18.0
Anal-fin base length	22.3	23.0
Anal-fin spine length	7.1	6.9
Longest anal-fin ray length	18.7	17.7
Caudal-fin length	27.3	27.0
Pectoral-fin length	24.8	25.2
Pelvic-fin spine length	6.2	7.3
Pelvic-fin ray length	22.0	22.2

Mouth terminal, oblique, inclined approximately 30° to horizontal body axis; posterior margin of upper lip reaching vertical through middle of pupil; posterior margin of maxilla reaching level of posterior margin of pupil. Anterior naris circular, with short membranous tube, anteroventral to posterior naris, just above anterior margin of upper lip; posterior naris circular, with very short tube, slightly anterior to center of eye. Teeth in upper jaw in 9 or 10 rows on each side, slender, conical; lower jaw with 5 or 6 rows on each side; teeth posteriorly smaller, teeth band narrowing to single row; anteriorly progressively larger and more widely spaced; anterior part of upper and lower jaws with 5 or 6 enlarged teeth. No teeth on vomer or palatines. Inner edges of lips papillose. Tongue tip blunt. Gill rakers small, short; 3 on upper limb, 8 on lower limb (including 2 and 3 rudimentary rakers in holotype and paratype, respectively) of first gill arch.

Cephalic pores and papilla rows (Fig. 3) well developed; pore pattern including a large pore (B’) adjacent to each posterior naris, two unpaired pores (C and D) at anterior and posterior interorbital regions, respectively, a pore (E) at dorsoposterior corner of orbit, three postocular pores (F, G and H’), and three preopercular pores (M’, N, and O’); K’ and L’ pores above operculum absent. Longitudinal papilla rows on lateral surface and ventrum of head (Fig. 3A, C): a single row below posterior margin of orbit; three rows across cheek; two rows extending anteriorly from preopercular-canal pore O’ along lower margin of preopercle to just behind anterior edge of lower lip; a vertically oriented row on opercle, curving horizontally. Longitudinal papilla rows on dorsal surface of head (Fig. 3B): a single oblique row adjacent to nasal; three pairs of short rows behind eyes on nape; two long rows above preopercle and opercle; two pairs of short rows on predorsal region.

Body scales mostly cycloid, becoming weakly ctenoid from about anterior part of second dorsal fin to caudal peduncle, and on base of caudal fin, increasing in size posteriorly; head and pectoral-fin base naked; prepelvic region with partially embedded scales (Fig. 3C); fins scaleless, except for three to four rows at base of caudal fin.

First dorsal-fin origin slightly posterior to pelvic-fin base; second dorsal fin close to but clearly separated from first dorsal fin. Dorsal-fin spines slender, flexible. In males, first spine relatively short, about equal in length to fourth spine; second and third spines longest, filamentous; fifth spine shortest. In females, first to fourth spines subequal, not prolonged, fifth spine shortest. Second dorsal fin longer than anal fin in both sexes. All segmented rays of second dorsal fin branched; tip of last ray reaching caudal-fin base when depressed in males, reaching to just before caudal-fin base in females. Anal-fin origin below base of first segmented ray of second dorsal fin; anal-fin height slightly less than that of second dorsal fin when expanded. Anal-fin spine slender, flexible; all segmented rays branched, last ray not reaching caudal-fin base when depressed. Caudal fin ovate. Expanded pectoral fins rounded, posterior margin reaching to below posterior end of first dorsal-fin base. Pelvic-fin origin slightly posterior to vertical through pectoral-fin base; pelvic-fin tip not reaching anus. Pelvic fins united medially by frenum between spines, and interradial membrane between innermost segmented rays; pelvic frenum thin, with smooth posterior margin.

***Color when fresh*** (Figs 1A, 2A). Body milky-white, scattered with numerous small yellow spots; five large radiant orange blotches along upper half of lateral body surface, first (faint) just behind opercle, second below middle of first dorsal-fin base, third and fourth below second dorsal-fin base, and fifth at caudal-fin base. Head white ventrally, purplish-white laterally, with several yellow marble-like spots from snout to opercle and nape; spots generally similar in size to those on base of second dorsal fin; largest spot elongate, below eye (above posterior portion of maxilla). Pupil black; iris golden, orangish dorsally and yellowish ventrally. First dorsal fin dark greenish-grey; membranes with long vertical or oblique semi-translucent white marks between first and third spines in males, and between fourth and sixth spines in females; small indistinct black spots distally on outer margin of membrane between third and fourth spines; numerous small yellow spots near base of posterior membranes. Second dorsal fin with semi-translucent white and dark membranes and 3–6 longitudinal rows of yellow spots. Caudal-fin membrane translucent, with 2 or 3 transverse rows of yellow spots anteriorly, and longitudinal yellow bands on posterior and ventral portions. Anal fin semi-translucent white and dark, with two narrow longitudinal yellow bands. Pelvic fin semi-translucent white and dark, with several yellow spots. Pectoral fin semi-translucent dorsally, white with yellow spots ventrally.

***Color when preserved*** (Figs 1B, 2B). Head whitish, generally with dark to brown marbled pattern. Body pale brown to milky-white, with four large dark grey net-like blotches (first blotch behind opercle present in fresh specimens, but not retained after preservation). First dorsal fin blackish, with semi-translucent white markings near fin base; large semi-translucent white marking between fourth and sixth spines (female only). Second dorsal, anal, pectoral, pelvic, and caudal fins uniformly semi-translucent, with dark brown pigmentation. Orange and yellow spots on head, body, and fins (in fresh specimens) all faded.

#### Distribution and habitat.

Currently known only off Banyuwangi, East Java, Indonesia. According to local fishermen (pers. comm.), the species inhabits sandy bottoms in shallow coastal waters. However, its ecological association with alpheid shrimps, typical of species of the genus *Tomiyamichthys*, has not been directly observed.

#### Etymology.

The specific epithet *oriens* is a Latin participle meaning “east” or “the rising sun.” The name refers to the radiant orange coloration of the living specimen, evoking the hues of sunrise. It also refers to the type locality of the species in Banyuwangi, East Java, a region popularly known in Indonesia as “The Sunrise of Java” due to its geographic position at the easternmost extremity of the island. Treated as a noun in apposition.

#### Remarks.

On the basis of segmented dorsal- and anal-fin ray numbers, the valid species of *Tomiyamichthys* fall into four groups: those with 12 segmented rays (six species); those with 11 segmented rays (three species); those with 10 segmented rays (eight species); and those with nine segmented rays (three species) (see Allen et al. 2020, 2023; Sato and Motomura 2025). The new species of *Tomiyamichthys*, described herein, having nine segmented dorsal- and anal-fin rays, belongs to the last group together with the Indo-West Pacific species *T. latruncularius* (Klausewitz, 1974), and two recently described species from West Papua and southern Japan, *T. eyreae* and *T. hyacinthinus* Sato & Motomura, 2025, respectively.

Within the group characterized by nine segmented dorsal- and anal-fin rays, *T. oriens* sp. nov. is most similar to *T. hyacinthinus*, both species being readily distinguished from *T. latruncularius* and *T. eyreae* by the presence of two elongated filaments (second and third spines) in the first dorsal fin of males (vs. three elongated filaments, on the second to fourth spines in *T. eyreae*, and the first to third spines in *T. latruncularius*).

Additionally, *T. oriens* sp. nov. can be distinguished from *T. eyreae* and *T. latruncularius* (based on characters and meristics from Allen et al. 2020) by having a greater number of pectoral-fin rays and gill rakers (18 and 3 + 8, respectively, in *T. oriens* sp. nov. vs. 17 and 2 + 3, respectively, in *T. eyreae*); more longitudinal scale rows (60 or 61 vs. 55 or 56 in *T. eyreae* and 47–49 in *T. latruncularius*); a higher number of transverse scale rows than in *T. eyreae* (15 vs. 13 or 14), but fewer than in *T. latruncularius* (18); and a partially scaled prepelvic region (vs. scales absent in *T. latruncularius*). *Tomiyamichthys oriens* sp. nov. is further distinguished from *T. eyreae* by its lateral body pattern: in living and fresh specimens, *T. eyreae* exhibits two rows of large, irregular white blotches, one along the upper body and the other along the ventral half, such a pattern being absent in *T. oriens* sp. nov.

*Tomiyamichthys oriens* sp. nov. appears to differ from *T. hyacinthinus* in the following combination of morphological characters (comparative data for *T. hyacinthinus* based on Sato and Motomura 2025), including a greater number of pectoral-fin rays (18 in the former vs. 17 in the latter), fewer branched caudal-fin rays (13 vs. 14 or 15), a greater number of longitudinal scales (60 or 61 vs. 45–48), and fewer gill rakers (11 vs. 12–13). Although the color pattern of *T. oriens* sp. nov. is very similar to that of *T. hyacinthinus*, particularly in the arrangement of spots and blotches on the head and body, it differs in several respects: yellow spots on lateral surface of head relatively larger (similar in size to those on second dorsal-fin base), but fewer in number (vs. smaller and more numerous in *T. hyacinthinus*); five bright orange blotches present along lateral body wall (vs. orangish-dark brown blotches); and a small, indistinct black spot present on membrane between third and fourth dorsal-fin spines (vs. large, distinct spot).

**Figure 4.**
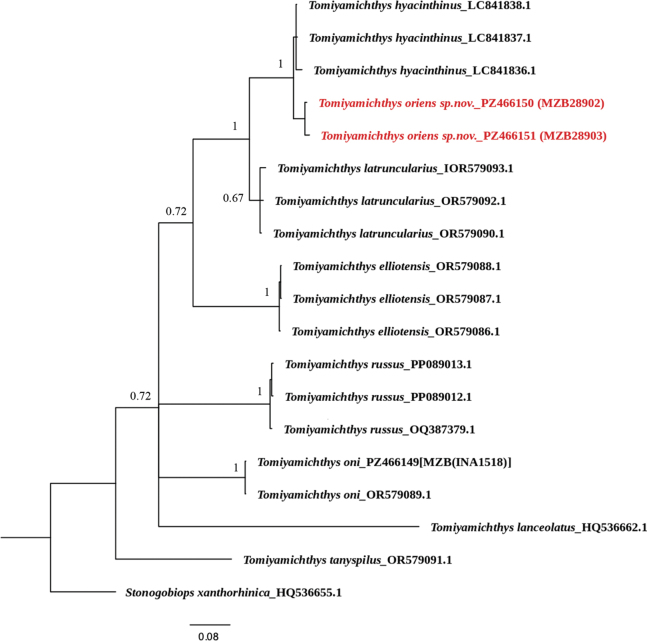
Bayesian phylogenetic tree inferred from partial mtDNA COI sequences. The analysis was performed using the HKY + Γ + I substitution model with a Markov Chain Monte Carlo (MCMC) run of 1,000,000 generations. Posterior probability values are indicated at the nodes. *Stonogobiops xanthorhinica* was used as the outgroup.

Genetic distances among eight species of *Tomiyamichthys*, calculated from partial sequences of the mitochondrial COI gene, reveal that *T. oriens* sp. nov. differs from *T. hyacinthinus* by 0.031 ± 0.006 and from *T. latruncularius* by 0.090 ± 0.012, *T. elliotensis* Allen, Erdmann & Dudgeon, 2023 by 0.167 ± 0.016, *T. russus* by 0.173 ± 0.016, *T. oni* by 0.177 ± 0.016, *T. lanceolatus* by 0.207 ± 0.018, and *T. tanyspilus* by 0.194 ± 0.017 (Fig. 4).

## Supplementary Material

XML Treatment for Tomiyamichthys oriens

## Additional information

### Artificial Intelligence (AI) use

The authors accept full responsibility for the content of the manuscript, including the disclosure of any use of AI.

No AI tools were used in the preparation of this manuscript.

### Author ORCIDs

K. Wibowo https://orcid.org/0000-0003-4465-3022

H.M. Pratiwi https://orcid.org/0000-0002-5883-3430

A.B. Dharmayanthi https://orcid.org/0000-0003-4635-6099

A. Priyadi https://orcid.org/0000-0001-8755-7239

R.V. Kusumah https://orcid.org/0000-0003-4477-8482

### Data availability

All of the data that support the findings of this study are available in the main text.
